# An orally available cancer drug AZD6738 prevents type 1 diabetes

**DOI:** 10.3389/fimmu.2023.1290058

**Published:** 2023-12-18

**Authors:** Norie Sugitani, Hannah R. Mason, Brian T. Campfield, Jon D. Piganelli

**Affiliations:** ^1^ Division of Pediatric Surgery, Department of Surgery, Pittsburgh, PA, United States; ^2^ Division of Pediatric Infectious Diseases, Department of Pediatrics, Pittsburgh, PA, United States; ^3^ University of Pittsburgh School of Medicine, University of Pittsburgh Medical Center (UPMC) Children’s Hospital of Pittsburgh, Pittsburgh, PA, United States; ^4^ Department of Endocrinology, Indiana University, Pittsburgh, PA, United States

**Keywords:** type 1 diabetes, prevention, T cell proliferation, DNA damage, ATR inhibition

## Abstract

Type 1 diabetes (T1D) affects three million Americans, with 80 new people diagnosed each day. T1D is currently uncurable and there is an urgent need to develop additional drug candidates to achieve the prevention of T1D. We propose AZD6738 (ATRi), an orally available drug currently in phases I and II of clinical trials for various cancers, as a novel candidate to prevent T1D. Based on previously reported findings of ATRi inducing cell death in rapidly proliferating T cells, we hypothesized that this drug would specifically affect self-antigen activated diabetogenic T cells. These cells, if left unchecked, could otherwise lead to the destruction of pancreatic β cells, contributing to the development of T1D. This work demonstrates that increasing the duration of ATRi treatment provides extended protection against T1D onset. Remarkably, 5-week ATRi treatment prevented T1D in a robust adoptive transfer mouse model. Furthermore, the splenocytes of animals that received 5-week ATRi treatment did not transfer immune-mediated diabetes, while the splenocytes from control animal transferred the disease in 10 days. This work shows that ATRi prevents T1D by specifically inducing cell death in self-antigen activated, highly proliferative diabetogenic T cells through the induction of DNA damage, resulting in the inhibition of IFNγ production and proliferation. These findings support the consideration of repurposing ATRi for T1D prevention.

## Introduction

Type 1 diabetes (T1D) is a chronic and incurable autoimmune disorder characterized by the destruction of pancreatic β cells by diabetogenic T cells, leading to insulin deficiency and hyperglycemia (1–3). The FDA approval of Teplizumab in late 2022 was an important first step to significantly delay T1D onset (4). However, the cost, the need of antibody infusion in children, as well as the heterogeneity of T1D necessitate the consideration of additional drug candidates to improve the delay of T1D onset and eventually prevent the disease. In this study, we propose an orally available ataxia telangiectasia mutated and Rad3-related (ATR) kinase inhibitor AZD6738 (ATRi), as a candidate for T1D prevention. ATRi is currently in phases I and II of clinical trials for various cancers (5–9). Previous research has shown that ATRi specifically induces cell death in actively proliferating T cells in the context of cancer (10). ATRi equally induces DNA replication damage regardless of cell types and species, but highly proliferative cells, experiencing DNA replication more frequently, are susceptible to cell death induced by ATRi (10, 11). Given that activated T cells proliferate up to 4 times daily, faster than most cancer cells (12, 13), and that self-antigen activated T cells targeting pancreatic β cells are the leading cause of T1D, we hypothesized that ATRi would delay the onset of T1D by inhibiting self-antigen activated, diabetogenic T cells. In the adoptive transfer mouse model, we show that 5-week ATRi treatment successfully prevents T1D. Furthermore, the animals transferred with the splenocytes of ATRi treated animals, containing reduced but viable T cells, did not experience diabetes for 38 days. Additionally, we found that in T1D, ATRi induces cell death in actively proliferating diabetogenic CD4^+^ T cells by promoting DNA damage accumulation, which results in proliferation inhibition. These findings strongly support ATRi as a promising candidate for T1D prevention.

## Methods

### Mice

NOD.BDC2.5 (NOD.Cg-Tg(TcraBDC2.5,TcrbBDC2.5)1Doi/DoiJ) and NOD.scid (NOD.Cg-Prkdc<scid>/J) mice were purchased from the Jackson laboratory. 8-12 weeks old animals were used. Both male and female NOD.BDC2.5 splenocytes were used and age- and sex-matched NOD.scid mice were used. All animals were housed in mouse cages at the animal facility at the Children’s Hospital of Pittsburgh. The University of Pittsburgh Institutional Animal Care and Use Committee approved the animal experiments performed.

### Antibodies

Anti-mouse CD28 (BD 553294), CD3_3_ (Fisher BDB553058), CD4 PE (Fisher BDB553730) at 1:250, CD8 APC (Fisher BDB553035) at 1:250, CD44 BV785 (BioLegend 103041) at 1:500, CD62L PE-Cy7 (BioLegend 104418) at 1:250, TCRβ A488 (BioLegend 109215) at 1:250, γH2AX A488 (BioLegend 613404) at 1:250, IFNγ (BD 551216), biotin IFNγ (BD 554410). All antibodies used for flow cytometry were diluted in 2% FBS in PBS.

### Chemicals

BD Violet Proliferation Dye 450 (CTV, Fisher BDB562158), Fixable Viability Dye eFluor™ 780 (Fisher 50-112-9035), Invitrogen™ eBioscience™ Foxp3/Transcription Factor Staining Buffer Set (Fisher 50-112-8857), Recombinant Mouse IFN-γ (Fisher BDB554587), Streptavidin-HRP (Invitrogen 434323), TMB substrate solution (Dako S1599), FxCycle™ Far Red Stain (ThermoFisher F10348), BDC2.5 mimotope RTRPLWVRME (InnoPep 3323-0100), EasySep™ Mouse Naïve CD4^+^ T Cell Isolation Kit (Stemcell technologies, Inc. 19765).

### Methods

Isolation, activation, and adoptive transfer of BDC2.5 CD4^+^ T cells into NOD.scid mice was performed as described previously (14). ATRi IFNγ analysis by ELISA (14), ATRi treatment, flow cytometry analysis as well as γH2AX staining (10) were performed as described previously. Details on replicates and statistical methods are described for each applicable data in the figure legends.

## Results

### ATRi treatment prevents T1D in the adoptive transfer mouse model

**Figure 1:**
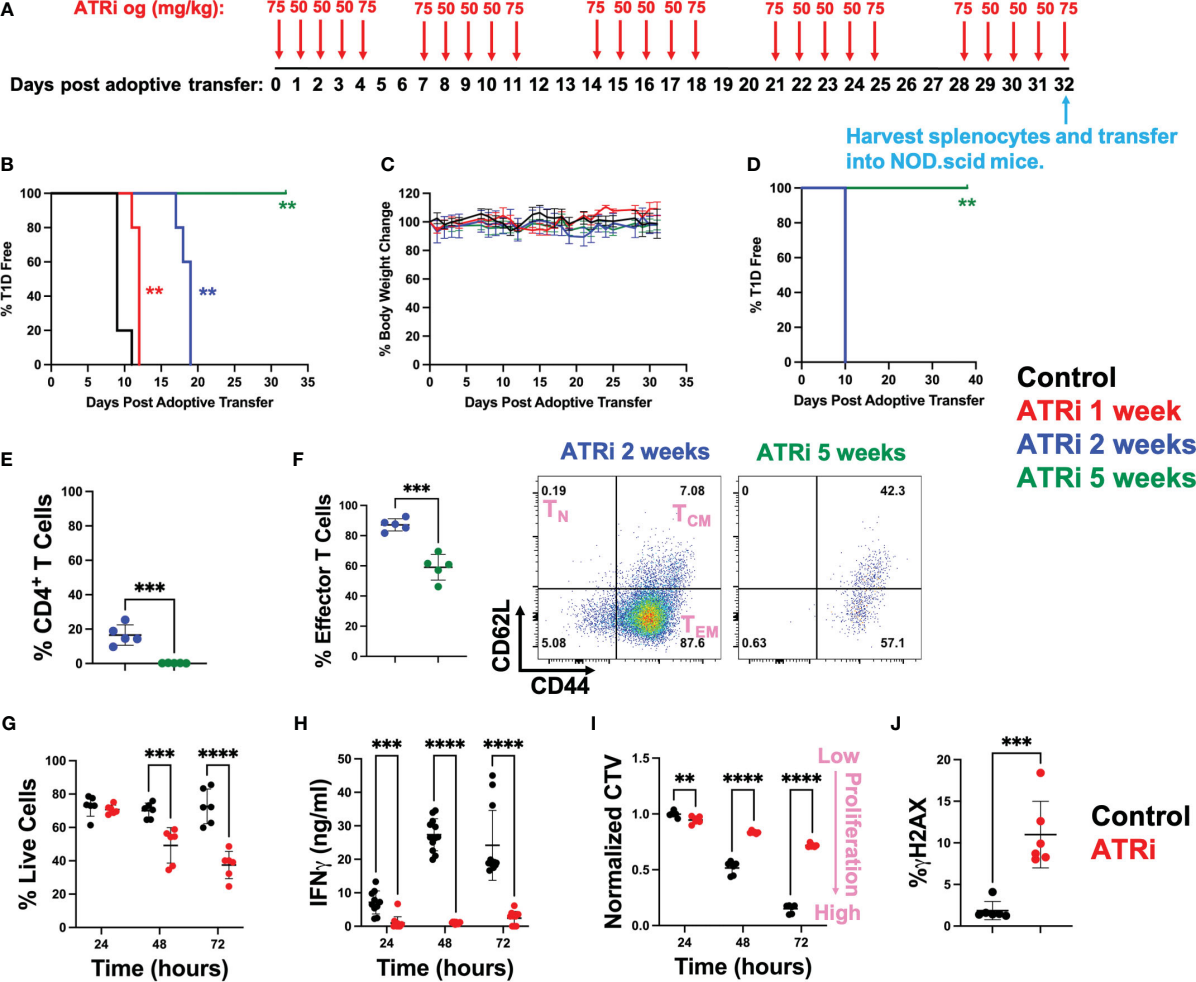
ATRi prevents type 1 diabetes by inhibiting proliferation of diabetogenic CD4^+^ T cells. **(A)** *In vivo* ATRi treatment schedule. NOD.scid mice were adoptively transferred with 2 million activated CD4^+^ T cells isolated from NOD.BDC2.5 mice. Mice were treated with vehicle (black) or indicated doses of ATRi by oral gavage (og) for 1 (red), 2 (blue), or 5 weeks (green). Type 1 diabetes incident **(B)** as well as % body weight change **(C)** of these mice were monitored. After 5 weeks of treatment, splenocytes from 2-week (blue, diabetic) or 5-week (green, diabetes free) ATRi treated splenocytes were harvested and transferred into a new set of NOD.scid mice. **(D)** T1D incident of newly transferred mice. **(E, F)** flow cytometry of transferred splenocytes. **(E)** population of CD4^+^ T cells within the splenocytes (in live gate). **(F)** % effector memory CD4^+^ T cells (left) and representative CD62L versus CD44 plot of 2-week or 5-week ATRi treated splenocytes (within live/CD4^+^ gate). T_N_: naïve T cells, T_CM_: central memory T cells, T_EM_: effector memory T cells. **(G-J)** Splenocytes from NOD.BDC2.5 mice were activated *ex vivo* with 0.25 μg/ml BDC2.5 mimotope and treated with vehicle (black) or 5 μM ATRi (red). **(G-J)** ATRi was given at the same time as activation. Survival **(G)**, effector function as indicated by IFNγ levels **(H)**, as well as proliferation indicated by the dilution of cell cycle violet (CTV) dye **(I)** at 24-, 48-, and 72-hours post activation are shown. Increased CTV dye dilution (decreased CTV intensity) represents increased proliferation. **(J)** Cells were treated for 4 hours starting at 48 hours post stimulation and DNA damage indicated by γH2AX levels are shown. **(B-F)** n=5, not significant (no asterisk), **: p<0.01, ***: p<0.001, ****: p<0.0001 by Mentel-Cox test **(B, D)**, Tukey **(C)**, or unpaired T-test **(E, F)**. **(G, I)** n=6 (three biological replicates with two technical replicate each). **(H)** n=10-12 (two biological replicates with six technical replicate each). **(J)** n=6 (two biological replicates with three technical replicates each). **(G-J)** not significant (no asterisk), **: p<0.01, ***: p<0.001, ****: p<0.0001 by unpaired T-test.

Diabetogenic CD4^+^ T cells were isolated from NOD.BDC2.5 mice, activated *ex vivo*, and adoptively transferred into NOD.scid mice that lack functional endogenous lymphocytes (14). Starting on the day of transfer, NOD.scid mice were treated with ATRi for up to 5 weeks by oral gavage as indicated in **Figure 1A**. We opted for a 5-day on/2-day off ATRi treatment cycle over 5 weeks to minimize potential adverse effects on the animals from extended treatment. While the 50 mg/kg dose was primarily used, the 75 mg/kg dose was employed before and after the 2-day off periods to accommodate the drug’s improved half-life with the increased dose (15). Both doses are commonly used in murine studies (5, 16, 17). The incidence of T1D, as determined by blood glucose levels over 300 for two consecutive days, was significantly delayed with an increasing length of ATRi treatment. 1 and 2 weeks of ATRi treatment resulted in approximately 2 or 8 days of delay, respectively (**Figure 1B**). Remarkably, none of the mice became diabetic when they received 5 weeks of ATRi treatment (**Figure 1B**). Furthermore, there was no significant weight loss or visible adverse effects observed in any group during ATRi treatment during the duration of the study (**Figure 1C**). After 5 weeks of treatment, splenocytes were collected from mice treated with ATRi for 2 weeks (diabetic) or 5 weeks (diabetes free), and re-transferred into a new set of NOD.scid mice. While mice transferred with diabetic splenocytes became diabetic in 10 days, the mice transferred with splenocytes from 5-week ATRi treated group remained free of diabetes during the time of study (over 1 month) (**Figure 1D**).

As expected, the splenocytes from 5-week ATRi treated mice had a limited number of CD4^+^ T cells compared to diabetic splenocytes prior to re-transfer (**Figure 1E**). However, ATRi did not completely eliminate CD4^+^ T cells in these splenocytes with 0.23 ± 0.11% CD4^+^ T cells left. Among these remaining CD4^+^ T cells, ATRi significantly reduced the effector memory population (**Figures 1F**, **S1**). 38 days post transfer in **Figure 1D**, spleens were harvested from the NOD.scid mice that received 5-week ATRi treated splenocytes. These splenocytes had 2.58 ± 0.24% live CD4^+^ T cells (**Figure S2**), confirming that T cells were successfully transferred into NOD.scid mice but did not transfer the disease within the duration of the study.

### ATRi specifically induces cell death in effector CD4^+^ T cell population by inhibiting proliferation, function and inducing DNA damage

To understand the mechanism by which ATRi inhibits diabetogenic effector CD4^+^ T cells, splenocytes from NOD.BDC2.5 mice were activated with BDC2.5 mimotope and treated with ATRi *ex vivo*. Consistent with the reduction of effector CD4^+^ T cells observed *in vivo*, ATRi treatment induced significant cell death *ex vivo* over time (**Figure 1G**). The effector function of these T cells, as determined by interferonγ (IFNγ) production, was inhibited by ATRi (**Figure 1H**). Cell trace violet (CTV) proliferation dye was employed to assess the effect of ATRi on proliferation of activated diabetogenic CD4^+^ T cells (**Figure 1I**). CTV dye is diluted as the cells divide. Therefore, decreased CTV intensity indicates increased proliferation. **Figure 1I** demonstrates that ATRi significantly decreased proliferation of activated CD4^+^ T cells. In addition, ATRi also elevated DNA damage levels (**Figure 1J**). We confirm that these effects are indeed through ATR function inhibition (as indicated by inhibition of Chk1 phosphorylation at S345) and it results in excess DNA replication origin firing (as indicated by the increase in hyper-phosphorylation of MCM4), consistent with previous studies (10, 11, 18) (**Figure S3**). Furthermore, while ATRi significantly reduced proliferation of CD4^+^ T cells, it did not affect activation of T cells which is consistent with ATRi studies in the context of cancer (10, 11, 18) (**Figure S4**).

## Discussion

The results of this study demonstrate, for the first time, that ATRi prevents T1D in the adoptive transfer mouse model by inducing DNA damage in actively proliferating diabetogenic CD4^+^ T cells, resulting in cell death within this population. The mechanism by which ATRi specifically induces cell death in highly proliferative diabetogenic effector T cells is consistent with observations in the context of cancer (10, 16, 19). In brief, ATR kinase inhibition leads to unscheduled DNA replication origin firing, resulting in depletion of nucleosides and an increase in DNA damage. However, it has been established that only cells that proliferate very rapidly are susceptible to ATRi-mediated cell death, leaving naïve T cells and other cell types, including proliferating cancer cells, viable (10). To confirm this in type 1 diabetes, the immediate next step is to test the efficacy of ATRi in the presence of naïve T cells in a spontaneous murine type 1 diabetes model. It has been reported that body weight is a dose limiting toxicity for AZD6738 due to potential effects in gastrointestinal tract (20). Despite targeting highly proliferative cells, the absence of adverse effects as well as significant weight loss observed with ATRi treatment, consistent with previous cancer studies (20), offers promise for its potential application in humans for T1D prevention.

Design of treatment strategy will be crucial when considering the application of ATRi to T1D prevention. In cancer clinical trials, myelosuppression has been associated with prolonged high-dose AZD6738 treatment (21, 22). However, a recent study has shown that both innate and adoptive immunity recover rapidly after short-course daily treatment (16). This study further described that prolonged daily ATRi treatment prevents recovery of antigen-specific T cell generation (16). While further in-depth research is necessary, the proposed regimen of intermittent mid-course daily ATRi treatment may help reduce the population and resurgence of diabetogenic T cells, while minimizing its effects on overall immunity. In short, that difference in cell types, activation status and proliferation rate are likely to result in differential susceptibility of each leucocyte to ATRi-induced cell death. Among leucocytes, activated T cells, B cells and neutrophils are the populations that has the potential to proliferate rapidly (23–25). Activated CD4^+^ and CD8^+^ T cells can divide up to 4 times daily, which, to the best of our knowledge, is the fastest proliferation rate reported among these lymphocytes (12, 13). Considering that self-antigen activated T cells are the major rapidly proliferating population in the individuals at risk of developing T1D, making them the major target for ATRi, we believe that fine-tuning of drug dose schedule can maximize preventative benefits while minimizing significant damage to other leucocytes.

Self-antigen-activated T cells against pancreatic β cells are the target of ATRi in T1D prevention. However, in certain situations, individuals at risk of T1D may also have actively proliferating T cells against other antigens, including those recently vaccinated, dealing with infections, cancer patients, or having other autoimmune diseases. To establish the ideal timing for initiating ATRi treatment in T1D prevention, these factors should be considered. In cases of vaccination or recent infections, a straightforward approach is to delay treatment until memory T cell populations develop or infections clear, supported by our study showing a milder impact of ATRi on central memory T cells compared to effector memory T cells (**Figures 1**F/S1). While numerous clinical studies have underscored the benefits of AZD6738 in cancer treatment, its use in patients with both cancer and T1D prevention requires cautious consideration. Extended daily ATRi treatment may impede the recovery of cancer antigen-specific CD8^+^ T cells crucial for cancer treatment. Hence, using ATRi in such patients demands thorough risk assessment and a carefully tailored treatment approach. Conversely, patients with other autoimmune diseases may potentially benefit from ATRi treatment due to its potential to reduce self-antigen-activated T cells.

Further studies are required to thoroughly assess the efficacy and safety of ATRi for T1D delay and eventual prevention in humans. That being said, considering its oral administration capacity, availability of human clinical data in cancer studies, and the successful prevention of the disease in a mouse model as demonstrated in this study, ATRi treatment should be considered as a potential preventive option for T1D in humans.

## Data availability statement

The original contributions presented in the study are included in the article/**Supplementary Material**. Further inquiries can be directed to the corresponding author.

## Ethics statement

The animal study was approved by University of Pittsburgh Institutional Animal Care and Use. The study was conducted in accordance with the local legislation and institutional requirements.

## Author contributions

NS: Conceptualization, Data curation, Formal analysis, Investigation, Methodology, Project administration, Supervision, Validation, Visualization, Writing – original draft, Writing – review & editing. HM: Data curation, Formal analysis, Writing – review & editing, Investigation, Validation. BC: Resources, Supervision, Formal analysis, Writing – review & editing. JP: Conceptualization, Funding acquisition, Resources, Supervision, Writing – review & editing.
