# Supplementary material for: An orally available cancer drug AZD6738 prevents type 1 diabetes
Source: Front Immunol. 2023 Dec 18;14:1290058. doi: 10.3389/fimmu.2023.1290058 (PMC10757955; doi:10.3389/fimmu.2023.1290058)
Supplement: Supplementary file 1 [file DataSheet_1.pdf]

Supplementary Figures

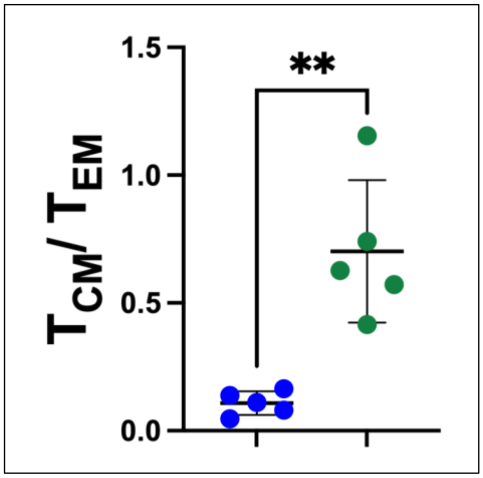

Figure S1: Ratio of Central Memory to Effector Memory CD4⁺ T Cells in 2-week (diabetic) or 5-week (T1D-free) ATRi treated splenocytes. n=5. **: $p<0.01$ by unpaired T-test.

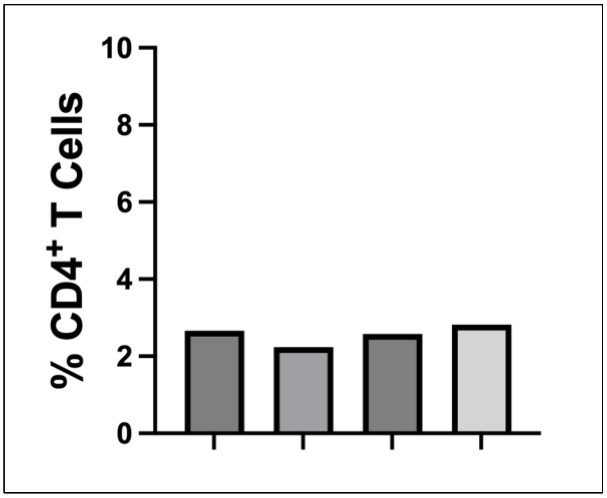

Figure S2: 5-week ATRi treated splenocytes transferred $CD4^+$ T cells in NOD.scid mice. 38 days post transfer of 2 million splenocytes from 5-week ATRi treated mice, spleens were harvested from each NOD.scid recipient mouse and percentages of live $CD4^+$ T cells were analyzed by flow cytometry. % $CD4^+$ T cells in live gate of each animal are shown.

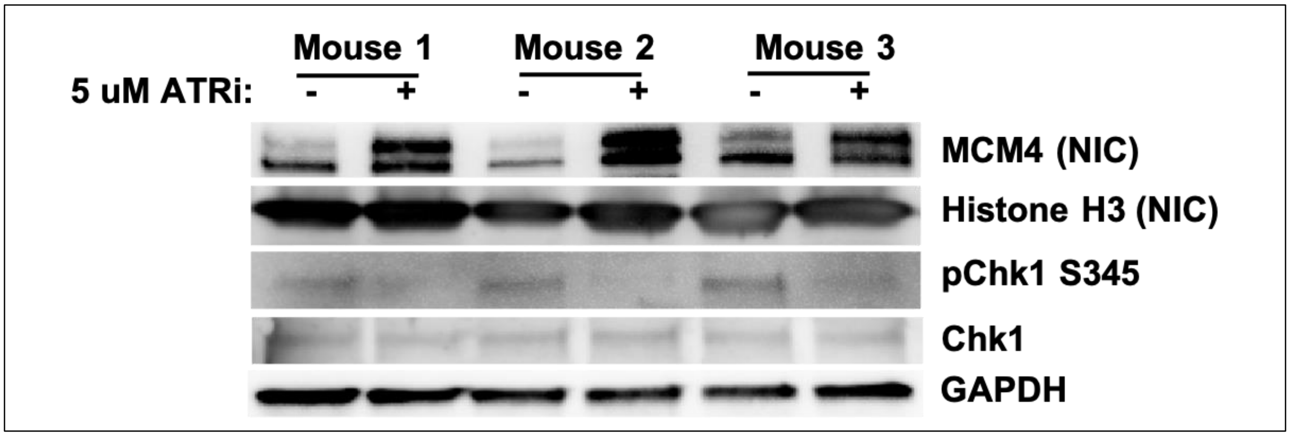

Figure S3: AZD6738 inhibits ATR activity and induces excess DNA replication origin firing in activated CD4⁺ T cells. CD4⁺ T cells were isolated from spleens of three BL/6 mice and activated *ex vivo*. 72 hours post stimulation, T cells were treated with vehicle or 5 μM ATRi for 1 hour. Harvested T cells were separated into lysate and nuclease insoluble chromatin (NIC) fractions and analyzed by Western blot. n=3.

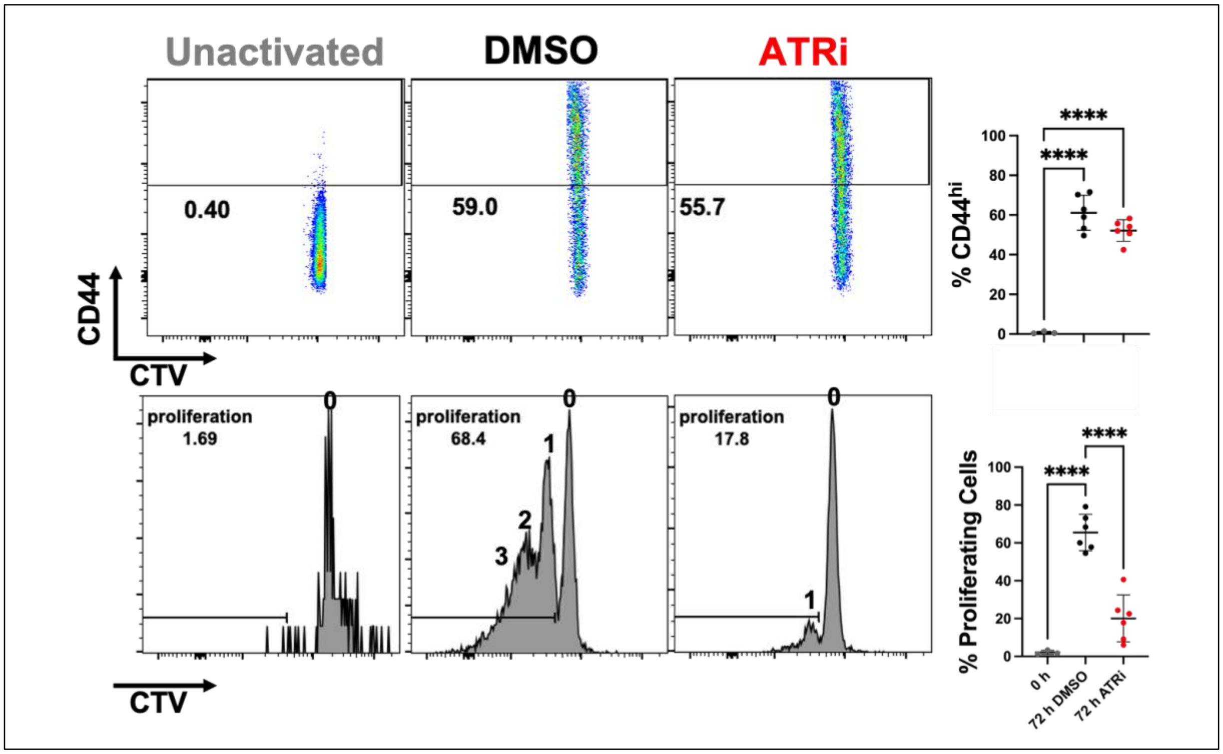

Figure S4: AZD6738 inhibits proliferation but not activation of CD4⁺ T cells. CD4⁺ T cells were isolated from spleens of three BL/6 mice and activated *ex vivo*. Starting at the time of activation, T cells were treated with DMSO or 5 μM ATRi for 72 hours. Activation (CD44^{hi}) and proliferation (dilution of CTV die, proliferation frequency indicated) were analyzed by flow cytometry. Top – CD44 vs CTV plots of 0 h (unactivated) or 72 h post stimulation with DMSO or ATRi treated cells within 0 proliferation gate (no CTV dilution). Bottom – CTV histogram of 0 h (unactivated) or 72 h post stimulation with DMSO or ATRi treated cells within CD44^{hi} gate. n=6 (three biological replicates with two technical replicates each). Not significant (no asterisk), ****: p<0.0001 by Tukey.

Supplementary Methods

Mice: C57BL/6 mice were purchased from the Jackson laboratory. Both male and female animals between 8-12 weeks old were used. All animals were housed in mouse cages at the animal facility at the Children's Hospital of Pittsburgh. The University of Pittsburgh Institutional Animal Care and Use Committee approved the animal experiments performed.

Antibodies: Rabbit anti-mouse Chk1 pS345 (Cell Signaling 2348S), Chk1 (ab32531), histone H3 (ab1791) at 1:1,000, MCM4 (ab12973) at 1:500, GAPDH (Millipore G9545) at 1:10,000. Goat anti-rabbit IgG (Thermo Scientific 31460) at 1:10,000.

Methods: $CD4^+$ T cells were isolated and activated from the splenocytes of C57BL/6 mice in the same manner as those from NOD.BDC2.5 mice. For Western blot (Figure S3), T cells were activated for 48-hours, removed anti-CD3 and anti-CD28 antibodies, and allowed to proliferate for an additional 24 hours. 72 hours post activation, T cells were treated with vehicle or 5 μM ATRi for 1 hour. Harvested cells were fractionated and analyzed by Western blot as described previously[1]. For Figure S4, T cells were treated with vehicle or 5 μM ATRi at the time of activation and analyzed by flow cytometry at 72-hour post stimulation. Details on replicates and statistical methods are described for each applicable data in the figure legends.

Supplementary Reference

1 Sugitani, N. *et al.* Thymidine rescues ATR kinase inhibitor-induced deoxyuridine contamination in genomic DNA, cell death, and interferon-alpha/beta expression. *Cell Rep* **40**, 111371 (2022). https://doi.org/10.1016/j.celrep.2022.111371
